# Identification of a prognostic 4-mRNA signature in laryngeal squamous cell carcinoma

**DOI:** 10.7150/jca.47557

**Published:** 2021-08-03

**Authors:** Cheng Zhang, Bin Shen, Xinwei Chen, Shang Gao, Xinjiang Ying, Pin Dong

**Affiliations:** 1 Department of Otorhinolaryngology-head and neck surgery, Shanghai General Hospital, Shanghai Jiao Tong University School of Medicine, Shanghai, China.; 2 Department of Otorhinolaryngology-head and neck surgery, Shanghai General Hospital, Shanghai, China.

**Keywords:** Laryngeal squamous cell carcinoma, prognosis, risk model, GEO, TCGA

## Abstract

**Background:** Laryngeal squamous cell carcinoma (LSCC) is one of the most common malignancy in the respiratory tract and could reduce the quality of life seriously like dyspnea, dysphonia and dysphagia. Moreover, 5-year survival rate has decreased over the past 40 years. This study was designed to identify mRNAs that related to prognosis in LSCC to enable early detection and outcome improvement.

**Methods:** Gene expression profiles from Gene Expression Omnibus (GEO) (GSE59102, GSE84957) and The Cancer Genome Atlas (TCGA) were analyzed to identify differentially expressed genes (DEGs) with the help of bioinformatics tools. Functional enrichment analyses including Gene Ontology (GO) and pathway analysis were carried out to investigate the role of those genes and underlying molecular mechanisms in LSCC. Cox's regression analyses (univariate, LASSO and multivariate in order) were utilized to identify DEGs related with patients' overall survival and a 4-mRNA-based prognostic risk score model was established. Univariate and multivariate Cox's regression analyses were then performed on LSCC data (90 patients left) to identify independent predictors of OS, including the signature and clinicopathologic variables. The prognostic value of the gene signature was further validated and the genes were analyzed by GEPIA to get pan-cancer expression profiles.

**Results:** 444 differentially expressed mRNAs (250 up-regulated, 194 down-regulated) were identified based on the threshold of fold change > 2 and adjusted p value < 0.05. Univariate Cox's regression analysis showed that high risk score (HR: 3.056, 95% confidence interval [CI]: 0.135-0.649, p<0.001) and female (HR: 0.296, 95% CI: 2.020-4.624, p=0.002) were associated with relatively poor prognosis. Further multivariate Cox's regression analysis indicated that risk score and gender were independent prognostic factors (p<0.05). The risk score model could stratify patients into high- and low‑risk groups, which presents significantly differential overall survival (p= 8.252e-04). The AUCs of 1-, 3- and 5-year OS were 0.724, 0.783 and 0.818, respectively.

**Conclusions:** Our study provides evidence that the four-mRNA signature could serve as a biomarker to predict prognosis in LSCC, especially in long-term.

## Introduction

Laryngeal cancer remains one of the most common tumors of the respiratory tract in which squamous cell cancer is known to be the major pathological type 1. There are estimated 177,422 new cases diagnosed and approximately 94,771 deaths reported globally in 2018 2. Despite advances in treatments, 5-year survival rate has decreased from 66% to 63% over the past 40 years 1. Although the clinical TNM staging, histopathological grading, history of cigarette smoking and heavy drinking will remain valuable, it is possible to acquire molecular information about host and tumor to optimize the management of laryngeal squamous cell carcinoma (LSCC) 3. Therefore, it is necessary to stratify disease-associated risks in patients early and to change treatment strategy, such as modality and intensity in multidisciplinary cancer management, as well as types of medicine administered in different risk groups.

With the increasing application of high-throughput sequencing technology, changes in genomics and transcriptomics have provided essential insights into the molecular-level characteristics of diseases. Many researchers are engaged in exploring potential biomarkers for predicting prognosis early in head and neck squamous cell cancer (HNSCC) patients, one of which is LSCC. Accumulating evidence has shown that mRNA signatures had potential value in predicting prognosis. In the recent studies, a 6-gene prognostic signature (PEX11A, NLRP2, SERPINE1, UPK, CTTN and D2HGDH) was established and area under the curve (AUC) of receiver operating characteristic (ROC) curve in 5-year overall survival (OS) was increased to 0.74 by drawing 4. Kaplan-Meier (K-M) survival analysis was used to verify that a signature (IGF1R, LAMC2, ITGB1, and IL-6) has an excellent association with poor survival contributed to radioresistance 5. In laryngeal cancer patients, the prognostic accuracy of the 5-gene signature (EMP1, HOXB9, DPY19L2P1, MMP1 and KLHDC7B) for OS at 5 years was 0.862 6. However, more research focused on the extensive category and subsite-specific transcriptional markers for prognosis combining multiple databases are rarely systematically compared and identified. Besides, cost-effectiveness yet to be taken into consideration in clinical practice.

Therefore, in this study we aimed to build a robust prognostic signature with minimal combinations of genes based on Gene Expression Omnibus (GEO) 7 and The Cancer Genome Atlas (TCGA) 8, which might be used to develop a fast detection kit. Subsequently, we identified four potential prognostic mRNAs and confirmed the integrated 4-mRNA signature as a novel prognostic biomarker that might help effectively predict overall survival of LSCC patients.

## Material and Methods

### Data acquirement and preprocessing

**Figure 1:**
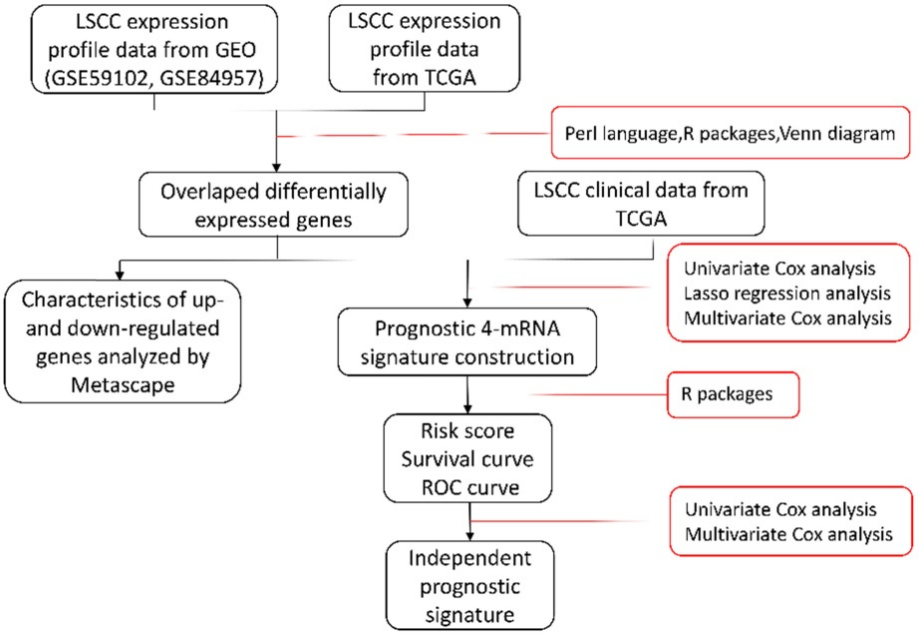
An overview of the gene model construction, red boxes represent methods used in every step. LSCC: laryngeal squamous cell carcinoma; GEO: Gene Expression Omnibus; TCGA: The Cancer Genome Atlas; ROC: receiver operating characteristic.

Samples selected for study based on the following specific criteria: diagnosed as LSCC; primary, untreated tumor with a source of matched normal tissue; informed consent from patients to donate part of their tumor samples. mRNA expression profile series matrix files of LSCC were retrieved from the GEO. Normalized mRNA expression values, in terms of level-3 fragments per kilobase of transcripts per million mapped reads (FPKM) and clinical meta data files (overall survival times and vital status) of patients with LSCC were downloaded from TCGA datasets via the Genomic Data Commons (GDC) Data Portal of National Cancer Institute and were used for subsequent survival-related analysis (details are listed in Table 1). Data were collected from September 2, 2019 to December 5, 2019. The flow chart of the entire study is displayed in Figure 1.

Strawberry-perl (version 5.30.1.1) was used to merge data sets of all samples from TCGA into one data set and convert probe IDs into gene symbols. As to TCGA, genome assembly Homo_sapiens.GRCh38.94.chr.gtf, which was downloaded from Ensembl, was used as a reference to map annotation. For GEO data, Agilent annotation files were utilized to perform the identifier conversion. Expression values were averaged when multiple probes corresponding to the same gene. Downstream analyses were conducted by using R (version 3.5.1).

### Differential expression analysis

The “limma” 9 package was executed for differentially expressed genes (DEGs) analysis in GEO, and Wilcoxon's rank sum tests were conducted by R function “wilcox.test”. DEGs were defined as fold change (FC) > 2 and adjusted *p* value (adj. P. val) < 0.05, where log_2_FC > 1 was regarded as up-regulation and log_2_FC < -1 as down-regulation. Visualization of the DEGs, in form of heatmap, were achieved by package “pheatmap”^10^ of R.

### Grouping of DEGs list

Web tool Venny (version 2.1) 11 was used to overlap up- and down-regulated gene lists with Venn's diagrams respectively.

### Construction of enriched ontology clusters

Aberrantly changed DEGs were submitted to Metascape 12, which incorporates a core set of default ontologies like Gene Ontology (GO) biological processes, Kyoto Encyclopedia of Genes and Genomes (KEGG) pathways, Reactome gene sets, Canonical pathways and so on, for biological processes and pathways analysis. Default settings were used, including the number of genes included in enrichment terms ≥ 3,* p* value ≤ 0.01 and minimum enrichment factor is 1.5.

### Prognostic mRNA signature construction

Combined expression quantity with clinical data, all DEGs genes associated with the OS were selected by using univariate Cox's proportional hazards regression. “Risky” genes (Hazard ratio [HR]> 1) and “protective” genes (0 < HR < 1) were selected in up- and down-regulated DEGs respectively. Subsequently, least absolute shrinkage and selection operator (LASSO) Cox's proportional hazards regression method (package “glmnet“) 13 and “survival“ 14, with a 10-fold cross-validation, were employed to acquire genes with proper weights. In order to facilitate the clinical application, the results were analyzed by using stepwise multivariable Cox's regression further and the best fitting COX model was selected based on the lowest Akaike's information criterion (AIC) 15.

We thus constructed a linear risk classifier model (formula as follows):


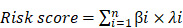


where 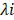 is the FPKM expression value and 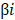 is corresponding regression coefficient of each individual gene.

### Identification of independent prognostic factors for LSCC

The independent predictors of OS (risk_score model, age, gender, cTNM_stage and pTNM_stage, treatment_type) were identified by univariate and multivariate Cox's proportional hazards regression.

### Performance of the mRNA signature

R package “pheatmap” 10 was utilized to draw risk related dot plots. Patients were divided into high- and low-risk groups according to the median risk score. The survival status of the two groups can also be displayed by dots with different colors separately. By running “survival” and “timeROC” 16 package in R, we obtained K-M survival curves. In addition, log-rank tests were applied to assess prognostic significance. The prediction performance of the model was presented by time-dependent ROC and evaluated based on the AUC in 1-, 3- and 5-year OS. All statistical tests were two-sided and *p*-values < 0.05 were considered statistically significant.

### Changes in genes expression of 33 cancer types

All genes in the signature were input into GEPIA 17 for differential expression analysis. Four-way analysis of variance (ANOVA) method was used to calculate differential expression between tumor and paired adjacent normal samples from TCGA and Genotype-Tissue Expression (GTEx) samples, where genes with |log2FC| values higher than 1 and q values lower than 0.01 are considered differentially expressed genes.

## Results

### Identification of DEGs

**Figure 2:**
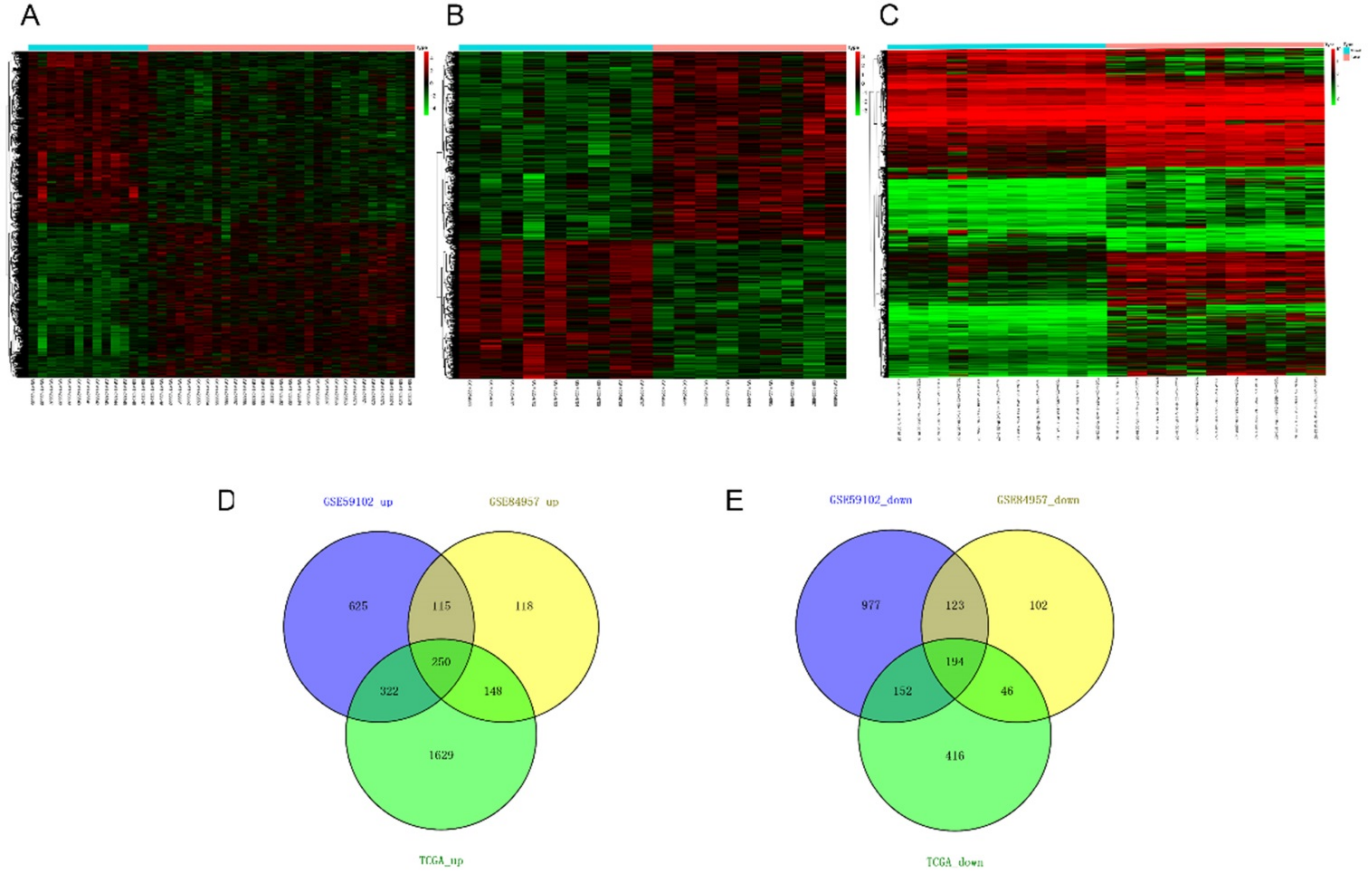
Heatmaps and Venn's diagrams of DEGs. (A-C) The heatmaps of 2758, 1096 and 3157 DEGs in GSE59102, GSE84957 and TCGA respectively (fold change > 2 and adjusted *p* value < 0.05), the blue bar represents that the tissue type is normal and blue is tumor. (D-E) The Venn diagrams indicate the overlapping of DEGs in three datasets mentioned above, where 250 up- and 194 down-regulated DEGs are showed. DEGs: differential expression genes.

Twenty nine cancer samples and thirteen margin samples of LSCC from GSE59102 18, 9 pairs of samples from GSE84957 19 and 11 paired RNA-sequencing (RNA-seq) data from TCGA were used to identify differential genes respectively (Table 2). According to the critical value mentioned in differential expression analysis, up- and down-regulated differential genes obtained from each datasets are taken into intersection analyses and drawn by Venn diagrams. The hierarchical-clustering heatmaps suggested differences in expression patterns between the two groups of samples in GSE59102, GSE84957 and TCGA (Figure 2A-2C). A total of 444 DEGs were identified, composed of 250 up-regulated mRNAs and 194 down-regulated mRNAs (Figure 2D-2E).

### Functional enrichment analysis

**Figure 3:**
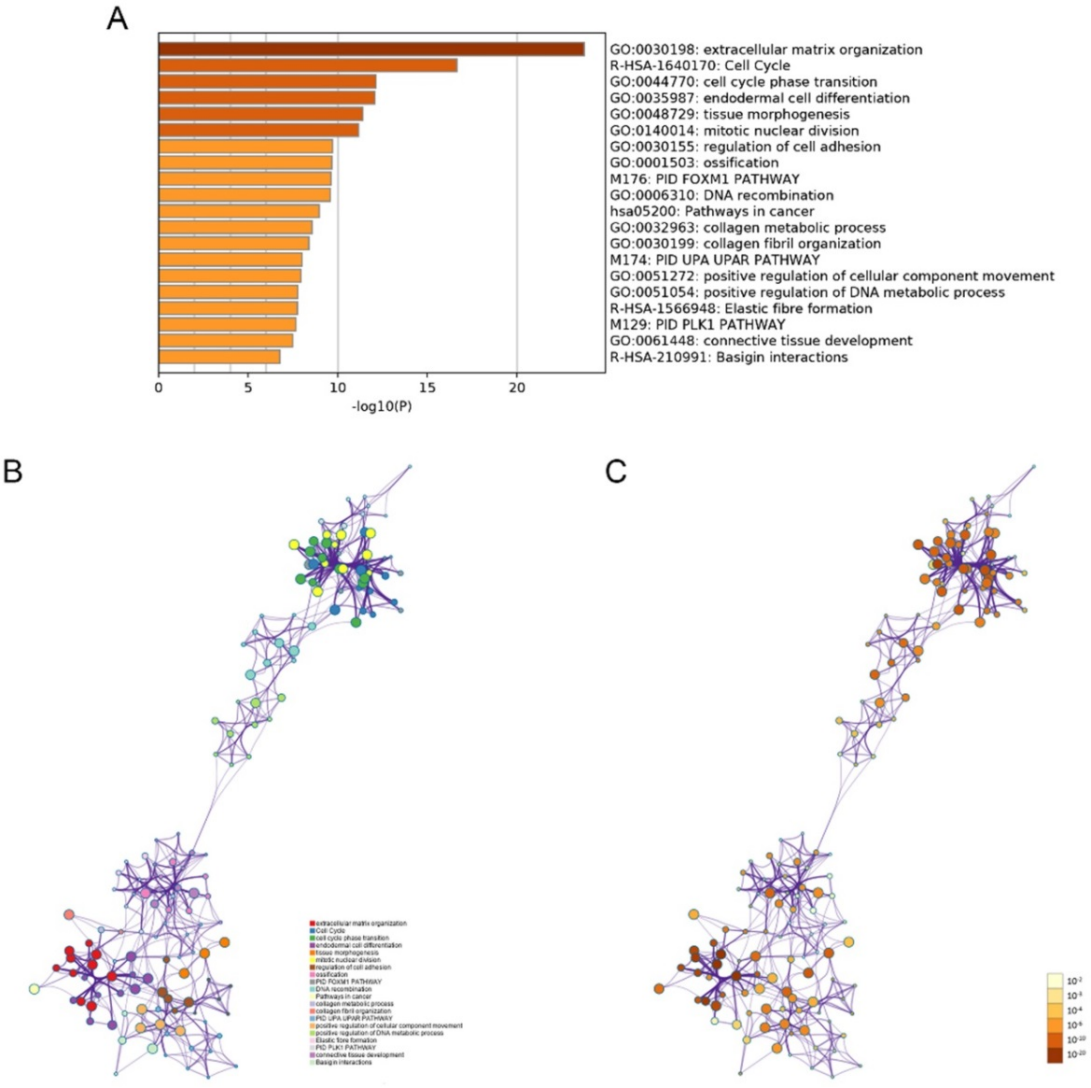
Functional enrichment analysis of up-regulated DEGs performed by Metascape. (A) Bar graph of top 20 non-redundant enrichment clusters and the color represents statistical significance. (B) Networks of enriched terms linked by edges representing Kappa similarity > 0.3. A Node represents each term and its size is proportional to the number of input genes fall into that term. Terms with the same cluster identity are marked corresponding color. (C) The same enrichment network as Figure B, darkness of the color indicated the *p*-value.

**Figure 4:**
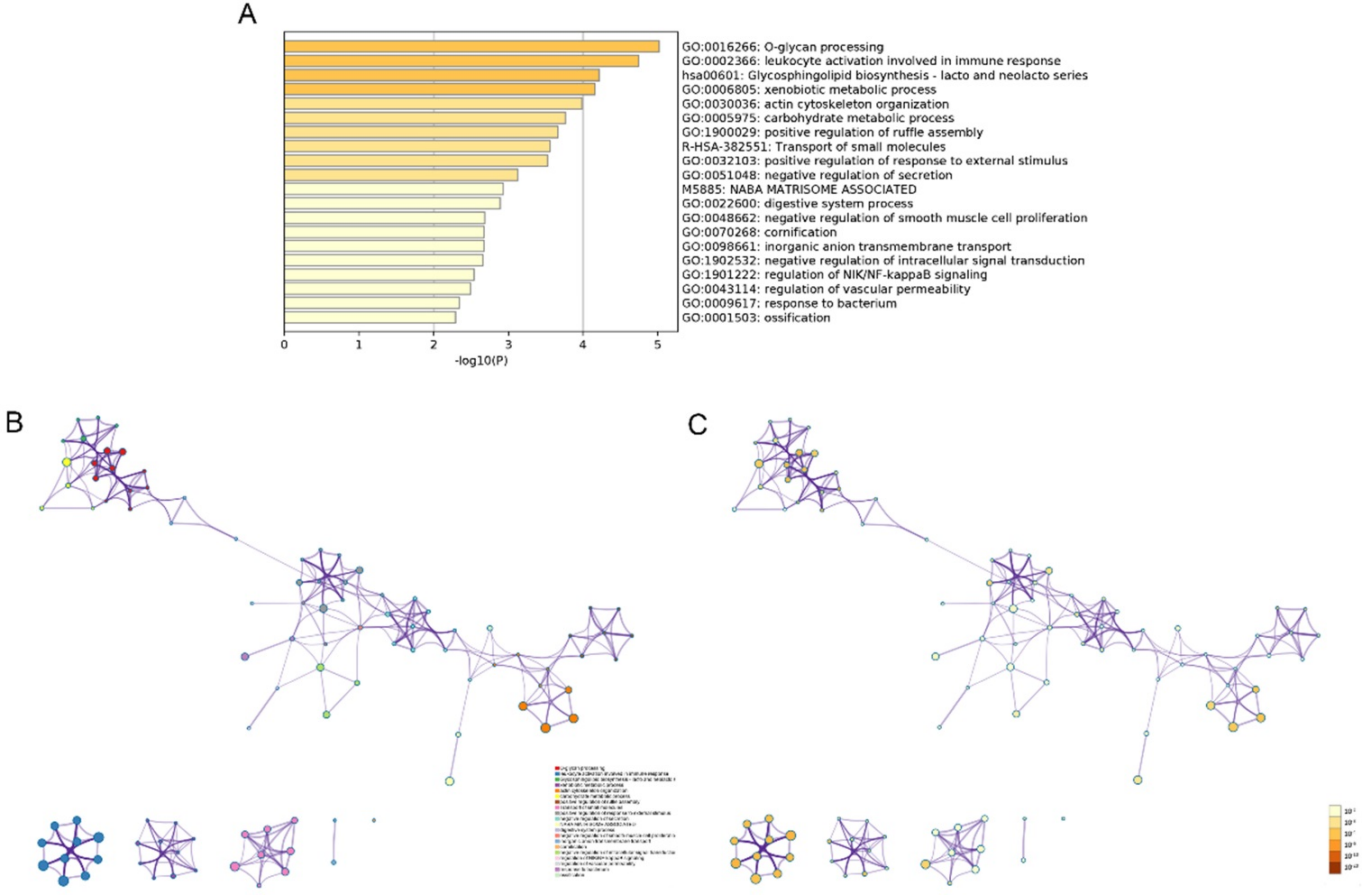
Functional enrichment analysis of down-regulated DEGs carried out by Metascape. (A) Bar graph of top 20 non-redundant enrichment clusters and the color represents statistical significance. (B) Networks of enriched terms linked by edges representing Kappa similarity > 0.3. A Node represents each term and its size is proportional to the number of input genes fall into that term. Terms with the same cluster identity are marked corresponding color. (C) The same enrichment network as Figure B, darkness of the color indicated the *p*-value.

The modulation-specific biological processes and pathways features of these up- and down-regulated DEGs were then analyzed. Results were visualized by bar graphs and clustering networks as shown in Figure 3-4. Among the top 20 output terms of up-regulated genes, enrichment is mainly found in extracellular matrix organization, cell cycle, cell adhesion and differentiation, and pathways in cancer, etc. Down-regulated genes are enriched in substance processing, leukocyte activation, metabolic processes, transport of substance, and regulation of signaling.

### Construction of 4-mRNA prognostic signature

**Figure 5:**
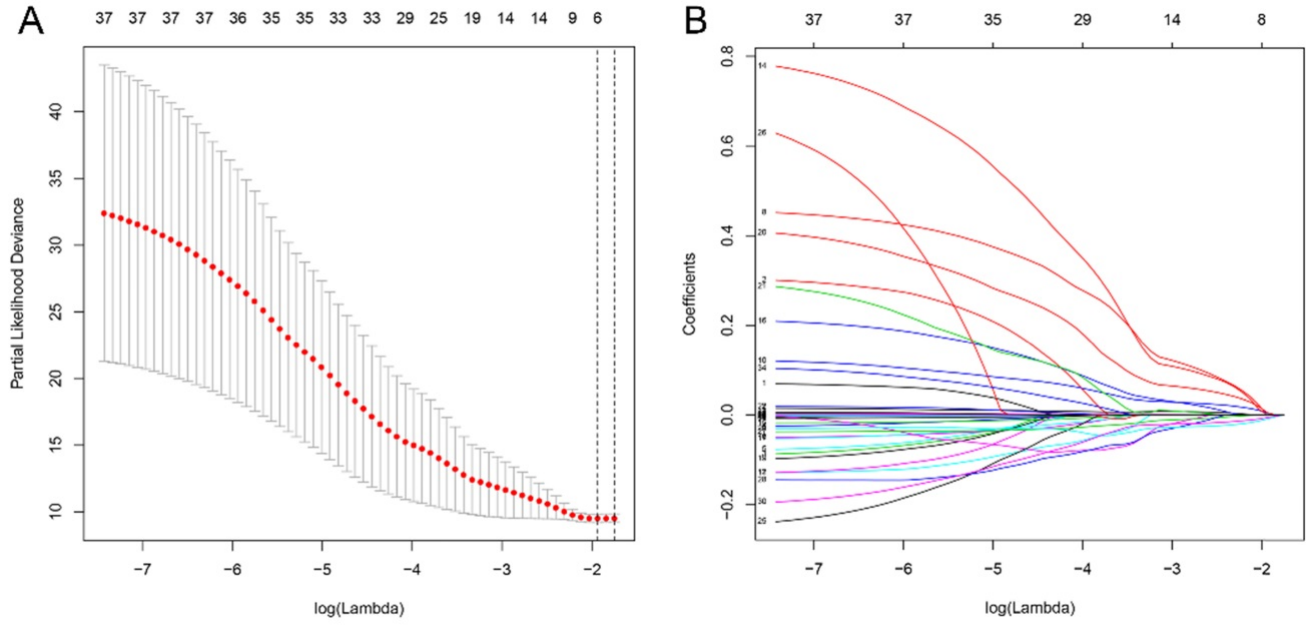
Analysis of 37 prognosis-associated DEGs analyzed by LASSO Cox's regression. (A) The left vertical bar indicates the minimum error, the right shows the largest value of 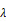 such that the error is within one standard deviation of the minimum. (B) Coefficient paths of the 37 DEGs that corresponded by value of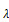. LASSO: least absolute shrinkage and selection operator.

All differential genes were analyzed by univariate Cox's proportional hazards regression. We obtained 32 OS-related genes in up-regulation gene sets and 7 in down-regulation gene sets (Table S1-2). In view of the classification criteria of “risk” and “protection” genes above, 2 up-regulated genes (CHTF18, SPC24) in tumor samples were excluded owing to their HR < 1. LASSO Cox's regression was performed on the recognized 37 DEGs. The optimal value (minimum error) of tuning parameter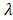, a constant controlling the degree of penalty, is 0.144 (Figure 5). Thus, 6 genes (STC2, ITGA5, AQP9, EPHX2, TCEA3, and MMP1) were selected out and further analyzed by multivariate Cox's proportional hazards regression to determine which one or more candidate genes could exhibit better predictive role. Consequently, we constructed a prognostic signature model based on the expression value of those mRNAs and their regression coefficients. The result is as follows: Risk score= 0.0494 × Expr (STC2) + 0.0866 × Expr (AQP9) + 0.0006 × Expr (MMP1) - 0.0721 × Expr (TCEA3).

### Univariate and multivariate Cox's regression analyses of the prognostic signature and clinical characteristics predictive of OS

**Figure 6:**
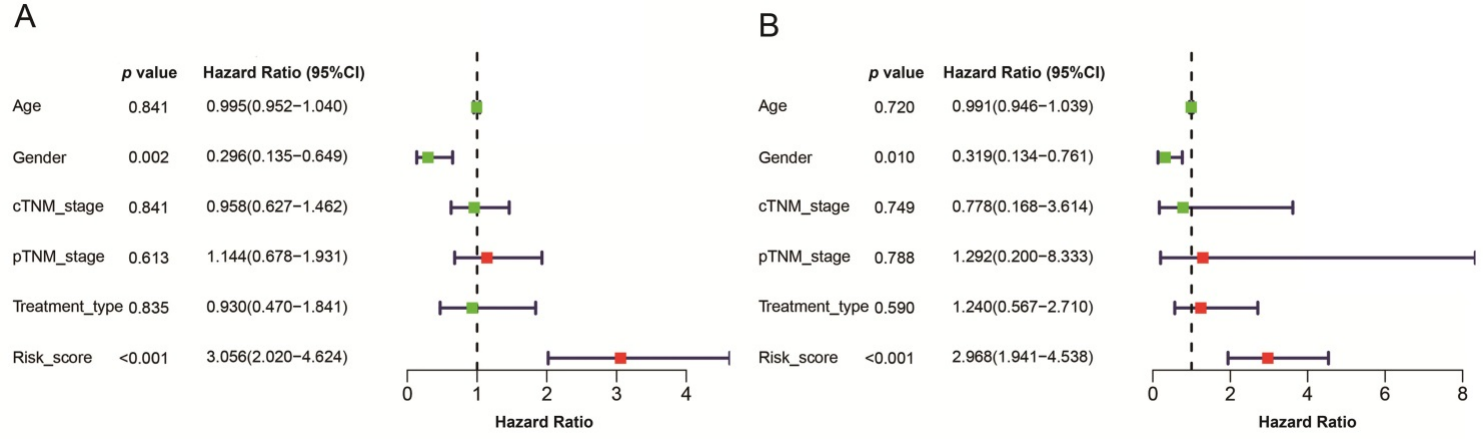
A model was identified based on univariate (A) and multivariate (B) analysis of independent predictors of OS. Bar lengths are hazard ratios (HR) for variables with 95% confidence intervals (95% CI), red boxes indicate HR>1, and the green boxes are the opposite. *p*-values < 0.05 were considered statistically significant.

Given that the AJCC cancer staging standards of laryngeal cancer in the 6^th^ edition 20 are the same as those in the 7^th^ edition 21, we excluded 4 cases clinical data of the 5^th^ edition 22 and integrated the former two editions. Furthermore, we also excluded 4 cases and 13 cases respectively for which cTNM and pTNM staging information was not available. Univariate and multivariate Cox's regression analyses were then performed on LSCC data (90 patients left) to identify independent predictors of OS, including the signature and clinicopathologic variables. Univariate Cox's regression analysis showed that risk score and gender were significantly associated with the prognosis of LSCC patients (Figure 6A). High risk score was associated with poor prognosis (HR: 3.056, 95% confidence interval [CI]: 0.135-0.649,* p*<0.001). Male were associated with relatively good prognosis (HR: 0.296, 95% CI: 2.020-4.624, *p*=0.002). Further multivariate Cox's regression analysis indicated that risk score and gender were independent prognostic factors (*p*<0.05) (Figure 6B).

### Estimations of the prognostic signature in the TCGA datasets

**Figure 7:**
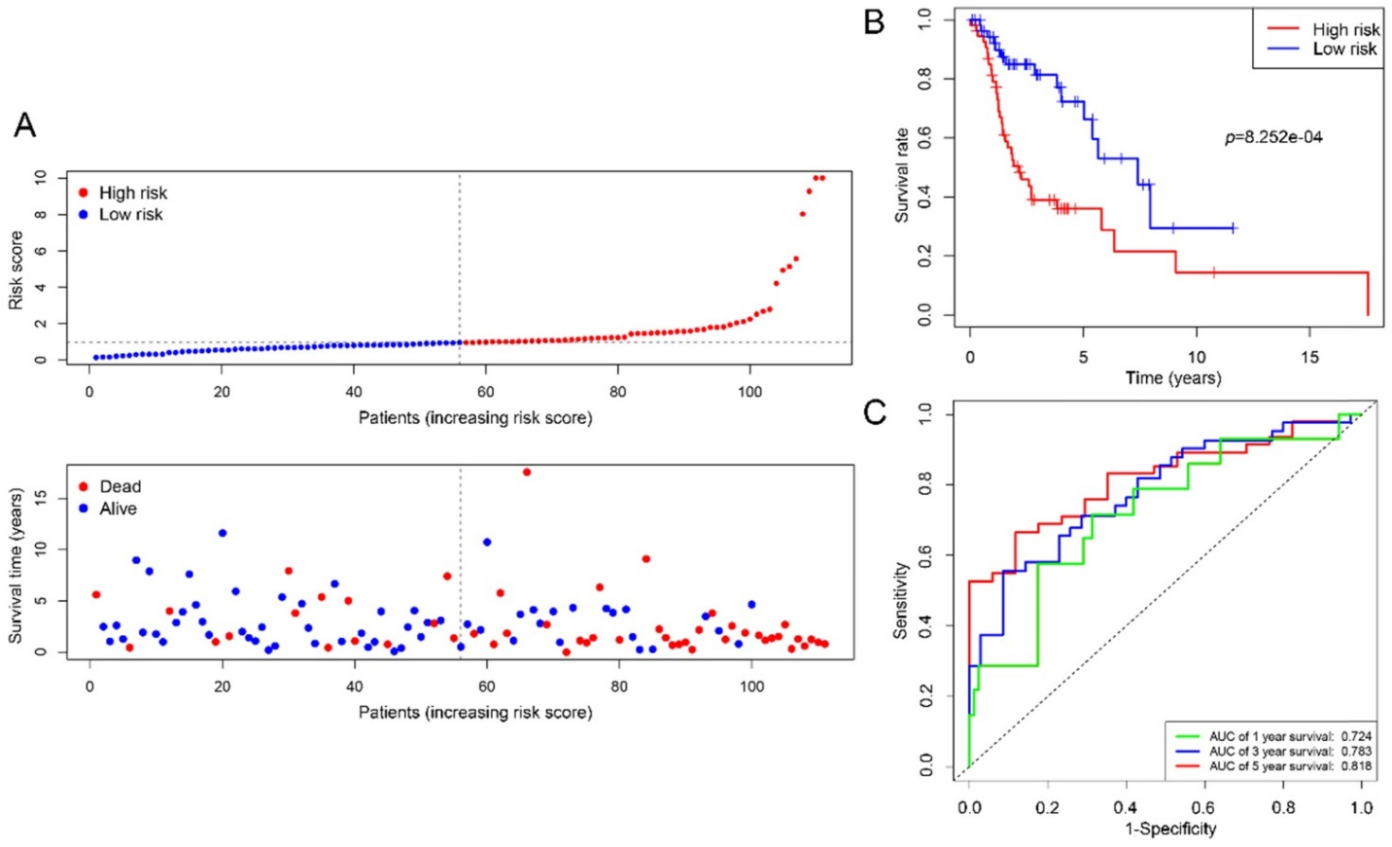
Estimations of the 4-mRNA prognostic signature in the TCGA datasets. (A) Vertical dotted line divides patients into low- and high-risk group based on the median risk score. Upper: curve of risk scores of all patients ranked in order of its increasing value. Lower: corresponding survival time with status for each patient. (B) Kaplan-Meier survival analysis of the 4-mRNA signature. (C) AUC values of 1-, 3-, and 5-years overall survival by drawing ROC curves. AUC: area under ROC curve.

We employed 111 of cases LSCC patients' clinical files from TCGA RNA-Seq to verify the prognostic value of the signature. The median risk score was used as a cutoff value to divide the cases into two types of risk groups, of which 63.6% (35/55) patients were deceased in the high-risk group and 26.8% (15/56) in the low-risk group (Figure 7A). K-M survival curves indicate that the signature could clearly distinguish different risk levels, where high-risk group had worse OS (*p*= 8.252e-04) (Figure 7B). Time-dependent ROC analysis showed that the AUC values of 1, 3 and 5 year survival were 0.724, 0.783 and 0.818 respectively (Figure 7C).

### Pan-cancer expression profile of genes in signature

MMP1 was over-expressed in all tumors where differential expression is exhibited. For instance, it is highly expressed in breast invasive carcinoma (BRCA), lung neoplasms and HNSC, etc. and lowly expressed in all normal tissues (Figure S1). STC2 was over-expressed in tumors such as colon adenocarcinoma (COAD), esophageal carcinoma (ESCA), glioblastoma multiforme (GBM) and HNSC, etc. and under-expressed in acute myeloid leukemia (LAML) and skin cutaneous melanoma (SKCM) (Figure S2). AQP9 was observed more down-regulated in all tumors found differential expressed like lung neoplasms, thyroid carcinoma (THYM) and up-regulated in ovarian serous cystadenocarcinoma (OV) and pancreatic adenocarcinoma (PAAD) (Figure S3). As to TCEA3, it was more under-expressed in tumors like HNSC, OV and PAAD, etc. (Figure S4).

## Discussion

Great efforts have been made to develop an optimal tool for predicting prognosis in LSCC patients, but no consensus has been reached. In this integrated analysis, candidates DEGs were selected by univariate and LASSO Cox's regression and were analyzed by multivariable Cox regression further to identify the best prognostic gene signature. Based on the expression value of these DEGs and their regression coefficients, a prognostic risk formula was constructed. The prediction accuracy of the model was analyzed by time‑dependent ROC and evaluated by the area under the curve. All AUC values are all greater than 0.7 and show an increasing trend as the survival time increases. High expression of “risky” mRNAs (STC2, AQP9 and MMP1) and low expression of “protective” mRNA (TCEA3) was found to be significantly associated with poor prognosis. Furthermore, K-M analysis confirmed their prognostic role in LSCC.

Taken together, the following are the issues we encountered and then addressed throughout the analysis. First of all, there are 11 tumor-adjacent samples that have their paired LSCC samples in the TCGA database, we thus used the 11 tumor samples instead of all to perform differential analyses. In this way, the inefficiency caused by a significant imbalance in the number of samples between groups is reduced 17. Secondly, the common significant DEGs were obtained from the intersection analyses of the results from multiple datasets sources, which could reduce systematic and random errors due to sequencing platforms and sampling, therefore, improves the robustness of the results and leads to the precise interpretation of molecular landscape of LSCC. Thirdly, we only selected data for LSCC to make the signature more specific. Fourthly, prognostic signatures based on multiple deregulated mRNAs have gained much attention recently and have shown their potential in prognosis prediction in different kinds of cancer 23, 24. Whereas, most single-gene biomarker researches focused more on basic experiments; ROC or other methods are rarely used to test their prediction accuracy. Last but not least, the basic concept of LASSO is that a penalty is used to shrink variable weights towards zero, with the result that small weights may get shrunken to zero and thus to prevent overfitting and improve model interpretability. Lambda is a hyperparameter that controls the strength of the penalty 25.

According to gene expression profiling studies, some aberrantly expressed mRNAs, such as HMGA2 26, FSCN1 27 and LAMA3 28, had been reported to be related to clinicopathological features in LSCC tissues. Considering that the Metascape could reduce the redundancy among ontology terms and monthly updates ensured by adopting a novel two-phase approach, we use the web-based portal to analyze the underlying biological processes and pathways of these DEGs involved in the genesis and development of LSCC.

In the results of the functional enrichment analysis of the 444 DEGs, MMP1 showed strong associations with biological processes related to extracellular matrix (ECM) organization, collagen metabolic process and pathways related to cancer, uPA/uPAR pathway and basigin interactions. Except for MMP1, the other three signature genes were not identified in the following top 20 enrichment terms. Matrix metalloproteinases (MMPs) represent a family of zinc-dependent proteinases, which can degrade ECM components, such as collagens and proteoglycans, and mediate tumor invasion and metastasis. Moreover, many MMPs identified in human are expressed and contribute to HNSCC progression. 29. Over-expression of MMP-1 has been found in all various cancers with differential expression 30, 31, which might indicate its underlying roles in those tumor phenotypes (Supplementary). Wang et.al found that Astrocyte elevated gene-1 (AEG-1) modulates the phosphorylation at serine 536 of the p65 subunit of NF-κB and enhances p65 binding to the MMP1 promoter, and subsequently increase the expression of the downstream gene in HNSCC 32. Some reports demonstrated that MMPs can be triggered by plasmin, which converted from plasminogen during urokinase plasminogen activator (uPA) /uPA receptors (uPAR) signaling pathway 33, 34. In addition, a study showed that co-localization of uPA with MMP-1, -2, -9 was observed in advanced epithelial ovarian primary tumors and metastatic lesions. 35. Kanekura and colleagues reported that coculture of basigin-expressing human malignant melanoma cells with dermal fibroblasts could induce the production of MMPs including MMP-1, MMP-2, MMP-3, etc. and induce invasion through a reconstituted basement membrane 36. Perturbation of basigin may have potential therapeutic uses in the prevention of MMP-2 and MMP-1-dependent cancer metastasis 37.

STC2 is a glycoprotein hormone involved in many biological processes, especially calcium and phosphate homeostasis, and it can also regulate the progression of malignant tumors 38. More recent researches revealed that downregulation of LINC00460 and HOTAIR could decrease STC2 via up-regulating microRNA-206 (miR-206) and promotes autophagy, proliferation, invasion and migration in HNSCC 39, 40. Moreover, a study showed that STC2 could upregulate the phosphorylation of AKT and enhance HNSCC metastasis via Snail-mediated increase of vimentin and the decrease of E-cadherin 41, which was proved that STC2 could activate PI3K/AKT signaling pathway by down-regulating miR-206 40. Note that STC2 was found over-expressed (Supplementary Figure) and might be directly regulated by HMGA2 at the transcriptional level in high-grade serous ovarian cancer 42. Furthermore, HMGA2 overexpression appeared to be a strong feature of larynx carcinoma 26.

Evidence showed that AQP9 played a critical role in the transmembrane transport of As_2_O_3_ and modulating arsenite sensitivity in leukemia 43, 44. In addition, patients treated with chemotherapy in AQP9 high expression subgroup showed significantly better disease-free survival in colorectal cancer, demonstrating AQP9 functions as a drug transporter and further sensitized tumor cells to chemotherapy drugs associated with RAS signaling activation 45. Recently AQP9 has also been found to play opposite roles in progression of different cancers. A report by Liao et al. identified that AQP9 could inhibit growth and metastasis of hepatocellular carcinoma cells via Wnt/β-catenin pathway 46. On the contrary, AQP9 would promote astrocytoma cell invasion and motility 47 and its increase in mRNA-level was significantly correlated with aggressive progression and poor survival in clear cell renal cell carcinoma patients 48.

As a member of the transcription elongation factor TFIIS family in vertebrates, TCEA3 was significantly downregulated in cancer tissues compared with paired normal tissues; its upregulation could induce apoptosis in gastric cancer, ovarian cancer and rhabdomyosarcoma cell lines 49-51.

Of course, all genes in our signature have been reported to be involved and played crucial roles in the development of many other tumors, these results could provide potential new insights for LSCC research. There are still some limitations in our study. Firstly, due to public databases and our center currently lack enough clinical samples, more predictive gene candidates could exist but were missed from our study of limited scope. Furthermore, in terms of the inconsistency of staging system annotations among samples and the absence of smoking history has further reduced the number of prognostic factors and samples to be comprehensively analyzed. Data used in the study only consisted of OS and still belong to the training set category. More external validation that covering risk score and other clinical variables in independent cohorts is required. Secondly, in addition to changes at the RNA level, changes of the protein and in-depth molecular-level mechanism could be investigated further.

## Conclusion

This study revealed deregulated mRNAs in LSCC, and discussed their possible roles in tumor progression. To our knowledge, the four-mRNA signature model has not been reported previously and could be promising to be a supplement to the individualized classification of LSCC patients, especially for the long-term survival. Nevertheless, further investigations of those DEGs and the signature are warranted.

## Supplementary Material

Supplementary figures and tables.

## Figures and Tables

**Table 1:** Clinicopathological characteristics of LSCC patients in TCGA mRNA (n=111)

Categories	n (%)
**Age at diagnosis**	
< 65	70 (63.06%)
≥ 65	41 (36.94%)
**Gender**	
Male	91 (81.98%)
Female	20 (18.02%)
**Race**	
Asian	1 (0.90%)
Black	19 (17.12%)
White	86 (77.48%)
Others	5 (4.50%)
**Staging system edition**	
5th	4 (3.60%)
6th	20 (18.02%)
7th	87 (78.38%)
**Clinical stage**	
I	3 (2.70%)
II	11 (9.91%)
III	26 (23.42%)
IV	67 (60.36%)
NA	4 (3.60%)
**Pathological stage**	
I	2 (1.80%)
II	9 (8.11%)
III	14 (12.61%)
IV	71 (63.96%)
NA	15 (13.51%)
**Treatment type**	
Pharmaceutical therapy	57 (51.35%)
Radiation therapy	54 (48.65%)
**Status**	
Alive	61 (54.95%)
Dead	50 (45.05%)

Abbreviations: LSCC: laryngeal squamous cell carcinoma; TCGA: The Cancer Genome Atlas; NA: not available.

**Table 2:** Overview of each datasets associated with LSCC from GEO and TCGA

Data source	Cases of tumor	Cases of normal	Platform	Scanned items	Clinical files
GSE59102	29	13	GPL6480	mRNA	No
GSE84957	9	9	GPL17843	lncRNA, mRNA	No
TCGA	111	12	NA	mRNA	Yes

Abbreviations: LSCC: laryngeal squamous cell carcinoma; GEO: Gene Expression Omnibus; TCGA: The Cancer Genome Atlas; GSE: GEO Series; GPL: GEO Platform; NA: not available.
