# Supplementary material for: Identification of a prognostic 4-mRNA signature in laryngeal squamous cell carcinoma
Source: J Cancer. 2021 Aug 3;12(19):5807–16. doi: 10.7150/jca.47557 (PMC8408111; doi:10.7150/jca.47557)
Supplement: Supplementary file 1 — Supplementary figures and tables. [file jcav12p5807s1.pdf]

Table S1. Thirty-two OS-related genes in up-regulation gene sets acquired by univariate Cox's proportional hazards regression analysis.

Gene symbols	HR	95%CI	*p* value
MMP12	1.004	1.000–1.007	0.034
LAMC2	1.001	1.000–1.002	0.043
MMP3	1.003	1.001–1.006	0.018
MFAP2	1.017	1.001–1.033	0.033
MMP1	1.001	1.000–1.001	<0.001
SERPINE1	1.002	1.000–1.004	0.021
RAB32	1.020	1.002–1.039	0.027
KLF7	1.101	1.022–1.186	0.011
NRIP3	1.212	1.003–1.465	0.046
MB21D2	1.094	1.007–1.190	0.034
LOXL2	1.019	1.001–1.038	0.042
HMGA2	1.063	1.011–1.117	0.016
PLAUR	1.027	1.000–1.054	0.048
TMEM158	1.021	1.006–1.036	0.006
TUBB3	1.294	1.057–1.585	0.013
INHBA	1.011	1.001–1.022	0.039
STC2	1.045	1.020–1.071	<0.001
ABL2	1.131	1.005–1.273	0.042
KIF7	1.219	1.013–1.466	0.036
TFRC	1.006	1.000–1.012	0.038
C3orf52	1.384	1.114–1.719	0.003
SERPINH1	1.007	1.001–1.013	0.034
FJX1	1.065	1.012–1.121	0.016
ITGA5	1.016	1.006–1.027	0.002
PFN2	1.007	1.001–1.013	0.025
C1QL1	1.143	1.040–1.256	0.006
AQP9	1.100	1.041–1.162	<0.001
FOXL2	1.159	1.027–1.308	0.017
LTBP1	1.012	1.002–1.023	0.024
MMP10	1.001	1.000–1.001	0.020
CHTF18	0.852	0.732–0.993	0.040
SPC24	0.841	0.730–0.970	0.017

Abbreviations: OS: overall survival; HR: hazard ratio; CI: confidence interval.

Table S2. Seven OS-related genes in down-regulation gene sets acquired by univariate Cox's proportional hazards regression analysis.

Gene symbols	HR	95%CI	*p* value
AGFG2	0.809	0.673–0.971	0.023
ACOX3	0.877	0.773–0.995	0.041
CPEB3	0.350	0.135–0.906	0.031
CFD	0.969	0.940–0.999	0.045
NFIA	0.799	0.641–0.995	0.045
EPHX2	0.816	0.695–0.958	0.013
TCEA3	0.904	0.842–0.970	0.005

Abbreviations: OS: overall survival; HR: hazard ratio; CI: confidence interval.

Figure S1

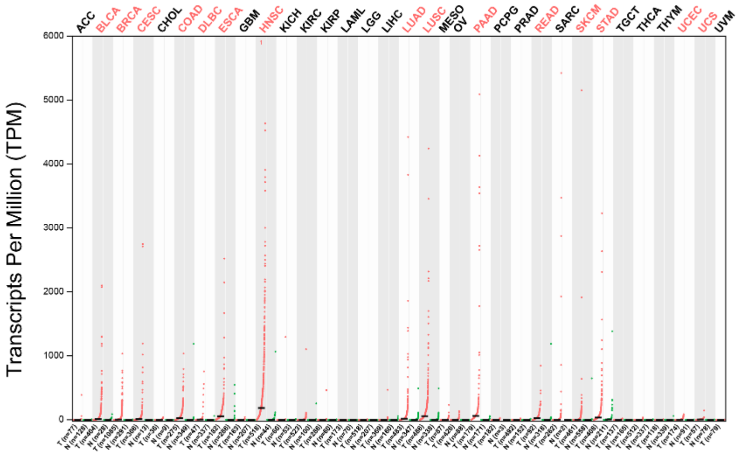

Figure S2

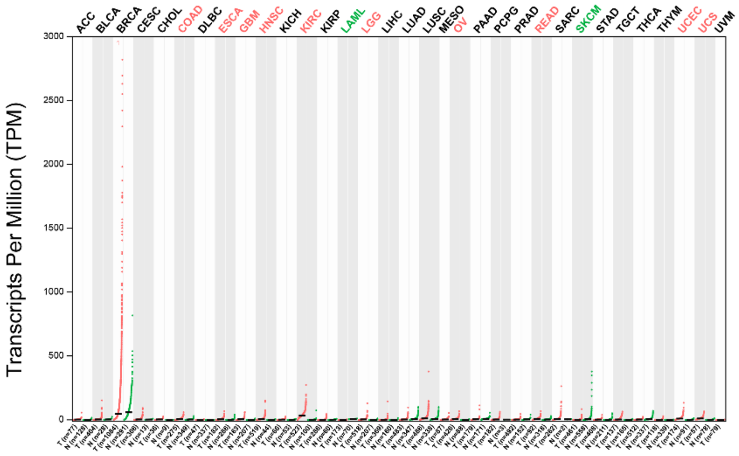

Figure S3

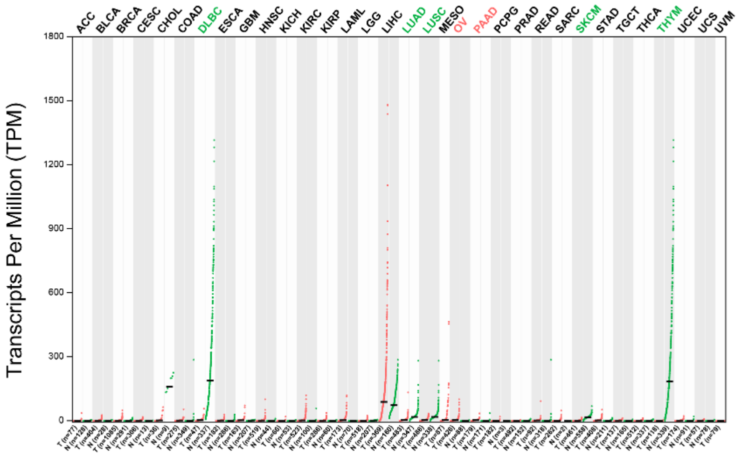

Figure S4

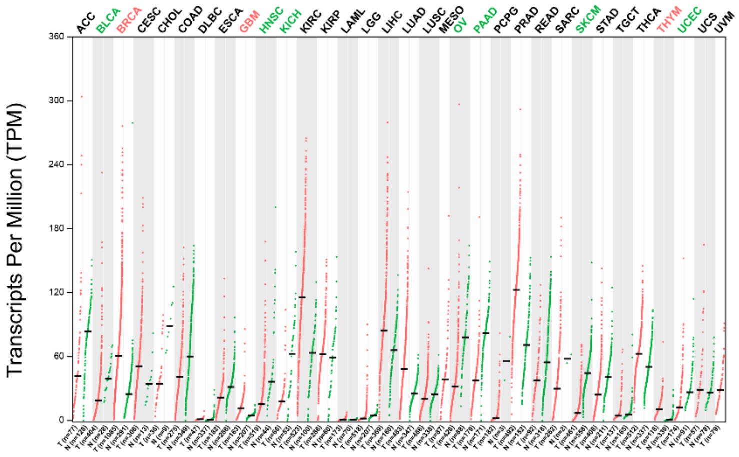

Figure legends

Figure S1-4. MMP1, STC2, AQP9 and TCEA3 expression profiles between tumor and paired adjacent normal samples from TCGA and GTEx in 33 cancers. GTEx: Genotype-Tissue Expression (fold change > 2 and q value < 0.01).
